# Development of a Novel Electrochemical Immunosensor for Rapid and Sensitive Detection of Sesame Allergens Ses i 4 and Ses i 5

**DOI:** 10.3390/foods14010115

**Published:** 2025-01-03

**Authors:** Huimei Li, Tian’ge Pan, Shudong He, Hanju Sun, Xiaodong Cao, Yongkang Ye

**Affiliations:** School of Food and Biological Engineering, Engineering Research Center of Bio-Process of Ministry of Education, Anhui Province Laboratory of Agricultural Products Modern Processing, Hefei University of Technology, Hefei 230009, China; 18713629852@163.com (H.L.); 15128774939@163.com (T.P.); sunhanjv@163.com (H.S.); xiaodongcao@hfut.edu.cn (X.C.)

**Keywords:** electrochemical immunosensor, allergen detection, oleosin, sesame seed, AuNPs–PEI–MWCNTs nanocomposite

## Abstract

Due to their lipophilicity and low content, the major sesame oleosin allergens, Ses i 4 and Ses i 5, are challenging to identify using conventional techniques. Then, a novel unlabeled electrochemical immunosensor was developed to detect the potential allergic activity of sesame oleosins. The voltammetric immunosensor was constructed using a composite of gold nanoparticles (AuNPs), polyethyleneimine (PEI), and multi-walled carbon nanotubes (MWCNTs), which was synthesized in a one-pot process and modified onto a glass carbon electrode to enhance the catalytic current of the oxygen reduction reaction. The oleosin antibody was then directed and immobilized onto the surface of the electrode, which had been modified with streptavidin (SPA), through the fragment crystallizable (Fc) region of the antibody. Under optimized conditions, the immunosensor exhibited a linear response within a detection range of 50 to 800 ng/L, with detection limits of 0.616 ng/L for Ses i 4 and 0.307 ng/L for Ses i 5, respectively. The immunosensor demonstrated excellent selectivity and stability, making it suitable for the quantification of sesame oleosins. The comparative analysis of various detection methods for sesame allergens was conducted, revealing that the immunosensor achieved a wide detection range and low limit of detection (LOD). Compared to traditional enzyme-linked immunosorbent assay (ELISA), the immunosensor successfully quantified the allergenicity potential of Ses i 4 and Ses i 5 in roasted sesame seeds at temperatures of 120 °C, 150 °C, and 180 °C. This innovative method offers a new perspective for the rapid quantification of sesame oleosins in foods and real-time monitoring of allergic potential, providing significant advancements in the field of food allergy detection.

## 1. Introduction

In recent decades, there has been a significant increase in public awareness regarding sesame seed (*Sesamum indicum* L.) allergies. As reported, the incidence of sesame allergy was increasing with a prevalence of sesame allergy in the general population, estimated to be between 0.1% and 0.2% [1]. By now, 7 sesame allergens, namely Ses i 1 to Ses i 7, have been identified, and most of allergens are water-soluble, such as 11S globulins, 7S vicilin-like globulin, and 2S albumin. Notably, two hydrophobic allergens from the oleosin family, known as Ses i 4 and Ses i 5, have received the most attention nowadays, as these kinds of allergens are difficult to be detected by prick tests and the immunological reactions in the aqueous system, but the oleosin allergens have been identified as the major allergens in sesame seed allergic patients by ELISA, isoelectric focalization (IEF) blotting, and sodium dodecyl sulfate–polyacrylamide gel electrophoresis (SDS-PAGE) blotting in a recent study [2].

Oleosin is classified as a hydrophobic, fundamental small molecular protein derived from plants with a molecular weight ranging from 15 to 24 kDa and is primarily found in the oil bodies to interact with the lipid and phospholipid fractions [3,4]. The structure of oleosin is made up of three unique parts as a hydrophobic domain with 72 amino acids in the middle, and on either sides are two amphiphilic helix-rich regions [5]. Notably, the core domain of oleosin is one of the longest hydrophobic domains identified to date, which not only complicates its solubility in water but also enhances the intricacy of sesame seed processing [6]. Specifically, during elevated-temperature roasting, the structure of oleosin would be profoundly changed because of the protein instability [7]. Due to the low content of oleosins in sesame and the difficulty of extracting it in an aqueous solution, only about one-third of sesame allergy patients can be diagnosed after consuming sesame through food challenge tests and auxiliary detection methods, such as skin prick tests (SPTs) and specific immunoglobulin E (sIgE) tests, and less allergy was confirmed to be induced by the sesame oleosins [8]. Moreover, although colorimetry immunoassay methods such as ELISA have been commonly used to measure the amount of food allergens present, the results would be disturbed in the oleosin detection because of the interference of lipid binding and/or high concentration of surfactants [9]. Moreover, organic solvents that are often used to facilitate the dissolution of oil bodies would interfere with the recognition between antigens and antibodies to reduce the efficiency of the immunoassay [10]. Since individuals with sesame allergies have an exceptionally low threshold for allergic reactions, potentially as low as 1 mg, a significant barrier would exist in the conventional immunological detection techniques, particularly in identifying low quantities of oleosin allergens in the oil body system. Therefore, there is an urgent need to develop more accurate detection techniques for sesame allergens, particularly Ses i 4 and Ses i 5.

Electrochemical sensors have garnered significant interest owing to their rapid analytical capabilities, excellent portability, high sensitivity, straightforward operation, elevated levels of automation, and ease of integration [11,12]. Furthermore, biosensors are known for their long-term stability and high reproducibility, and have been widely used as promising clinical tools. In previous studies, the biosensors have been employed in the detection of allergens from the oil crops of peanuts and hazelnuts [13,14]. In addition, label-free electrochemical biosensors have been developed for the detection of lipid-associated proteins, such as low-density lipoprotein (LDL) with high sensitivity and specificity, demonstrating that electrochemical biosensors could be successfully applied in the detection of oil bodies. Thus, electrochemical immunosensors combining the high sensitivity of chemical analysis with the precise selectivity of immunotechnology might be developed for oleosin allergen detections. Based on the specificity of the antigen–antibody interaction, anti-Ses i 4 and anti-Ses i 5 antibodies could be immobilized on the electrochemical platform, enabling specific recognition and rapid response to sesame oleosins, respectively. However, there are still no reports yet.

In the manufacturing process of immunosensors, the selection of materials would be a critical step that would significantly influence the overall performance. Advanced nanomaterials have been extensively utilized in research to enhance the analytical capabilities of electrochemical immunosensors. Emerging nanomaterials, including carbon nanomaterials, metal nanomaterials, and conductive polymers, have exhibited considerable potential for electrode modification [15]. Notably, double-walled carbon nanotubes (MWCNTs) have gained prominence in the realm of electrochemical immunosensors due to their exceptional electrical conductivity, large surface area, and favorable chemical reactivity, as evidenced by their successful application in the detection of aflatoxin B1 (AFB1) in peanuts [16]. Metal nanomaterials, particularly gold nanoparticles (AuNPs), have been also widely employed in this domain. AuNPs are highly regarded for their excellent biocompatibility, efficient electrocatalytic activity, and substantial surface area [17]. Despite the challenge posed by MWCNT aggregation in aqueous environments, the incorporation of polyethyleneimine (PEI), a highly water-soluble polyelectrolyte, has been shown to effectively stabilize carbon nanotubes and mitigate their aggregation. Additionally, the inclusion of PEI can enhance the binding efficiency of AuNPs [18]. Another challenge in the manufacturing of immunosensors would be the effective immobilization of antibodies. The selection of an appropriate immobilization method can significantly influence the binding efficiency of the antibody–antigen interaction, which subsequently affects the overall success of AuNP immunoassays [19]. Physical adsorption has been widely employed for antibody immobilization, wherein antibodies are securely adsorbed onto conventional solid supports, such as polystyrene, through hydrophobic and electrostatic interactions. While this method is relatively straightforward, it would result in a random orientation of the immobilized antibodies, which may lead to denaturation or displacement during subsequent washing procedures [20]. Consequently, utilizing immunoglobulin-binding Staphylococcus protein A (SPA) as an adsorbent may present a more advantageous alternative, which could offer a reliable means of achieving oriented antibodies by binding to the Fc region, and would enhance the antibody–antigen interaction [21]. Moreover, since the immobilization process does not necessitate antibody modification, the bound antibodies can maintain their full binding capacity. Therefore, in comparison to immunoassays employing conventional methods, such as random covalent immobilization, those that utilize antibody-binding proteins for antibody immobilization generally demonstrate superior sensing capabilities [22].

The current study aimed to explore a unique nanocomposite electrochemical sensor technology utilizing AuNPs–PEI–MWCNTs for the accurate detection of sesame allergens, notably Sesi 4 and Sesi 5. A comprehensive evaluation of sensors was involved for sensitivity, linearity, accuracy, and stability. Additionally, roasted sesame samples were applied to assess the accuracy of the immunosensors by comparing the performance with current commercial ELISA kits. This study will contribute to the advancement of nanomaterials in electrochemical biosensors and improve the accurate quantification of sesame allergens, providing a reliable basis for the safe production of sesame products.

## 2. Materials and Methods

### 2.1. Material

White sesame seed (*Sesamum indicum* L.) was obtained from Anhui Yushan Sesame Co., Ltd. (Anhui, China). Chloroauric acid (HAuCl_4_, 99.0%), potassium ferricyanide (K_3_[Fe(CN)_6_], 99.0%), potassium ferrocyanide (K_4_[Fe(CN)_6_], 99.0%), and polyethyleneimine (PEI, ≥99.0%) were purchased from Sinopharm Group Chemical Co., Ltd. (Shanghai, China). Carboxylated multi-walled carbon nanotubes (MWCNTs, ≥99.0%) were purchased from Xianfeng Nanomaterial Technology Co., Ltd., (Nanjing, China). Recombinant SPA was purchased from Solaibao Technology Co., Ltd., (Beijing, China). Phosphate-buffered saline (PBS) powder was purchased from ServiceBio Co., Ltd., (Wuhan, China) and dissolved in ultrapure water to prepare a 0.1 mM PBS solution. The Sodium dodecyl sulfate (SDS) was purchased from Biofroxx Co., Ltd., (Guangzhou, China). The ELISA kits, polyclonal antibodies prepared by immunizing rabbits with standard antigens of Ses i 4 and Ses i 5, and the standards of Ses i 4 and Ses i 5 were all purchased from Moshak Biological Co., Ltd. (Wuhan, China). All other reagents were of analytical grade and were used directly without further purification. Ultrapure water was used throughout the experiments.

### 2.2. Synthesis of AuNPs–PEI–MWCNTs Nanocomposites

**Figure 1:**
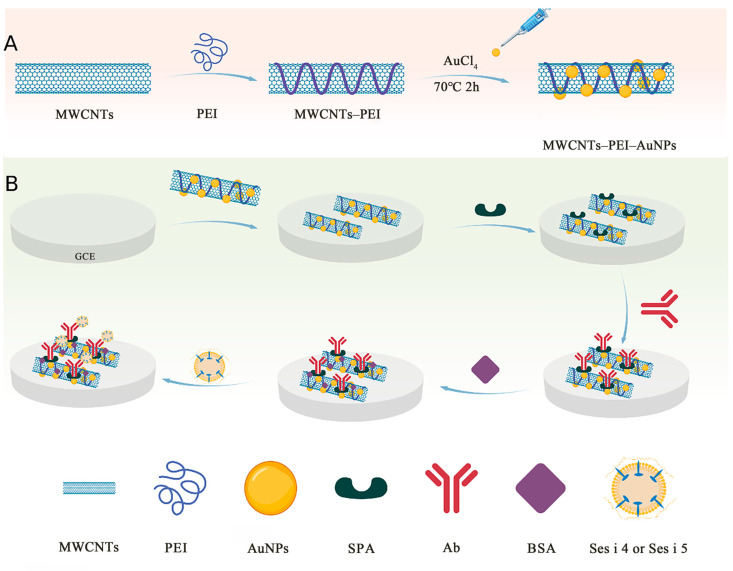
Schematic illustration for (**A**) synthesis of AuNPs–MWCNTs–PEI nanocomposite, (**B**) fabrication steps of the immunosensor for the determination of Ses i 4 or Ses i 5.

A primary method for the synthesis of AuNPs–PEI–MWCNTs nanocomposites was outlined by Sun (2019) with some modifications [23]. Specifically, 1 mg of MWCNTs and 25 mg of PEI were weighed and dissolved in 4 mL of ultrapure water with ultrasonic dissolution (Kunshan Chaosheng Co., Ltd. Shanghai, China). To generate AuNPs, 1 mL of HAuCl_4_ (10 mg/mL) was added to the mixture once it had been completely dissolved and dispersed. The mixture was then heated in a water bath at 70 °C for 2 h. The sample was then cooled to room temperature and centrifuged for 10 min at 9000× *g*. The purple-red supernatant was decanted, and the residue was re-dissolved using ultrapure water. The process was repeated three times until the supernatant no longer displayed a purple-red color. Figure 1A illustrates the synthesis procedure, detailing the steps that led to the formation of the nanocomposite material.

### 2.3. Fabrication of the Immunosensor

The glassy carbon electrode (GCE) was sequentially polished with 1.0 μm, 0.3 μm, and 0.05 μm alumina (Al_2_O_3_) slurries and then cleaned with a 50% (*v*/*v*) ethanol solution, followed by drying with nitrogen. Two milligrams of AuNPs–PEI–MWCNTs complex were mixed with 1 mL of ultrapure water and dispersed completely. Then 5 μL aliquots of the solution were applied dropwise to the center of the GCE and allowed to air-dry at room temperature. Next, 10 μL SPA (2 mg/mL in 10 mM PBS, pH 7.4) was dropped onto the electrode and was immobilized for 2h. Afterwards, 5 μL of Ses i 4 or Ses i 5 polyclonal antibody (2 mg/mL in 10 mM PBS, pH 7.4) were added, respectively, and incubated at 37 °C for 90 min. Once the incubation was complete, 10 μL of BSA (1%, *w*/*v*) was added for 1 h to the electrode surface to block excess active sites. Figure 1B shows the immunosensor fabrication method. Finally, the electrodes were immersed in a [Fe(CN)_6_]^3−/4−^ (5 mM) solution with 0.1 M KCl for cyclic voltammetry (CV) analysis.

### 2.4. Characterization of AuNPs–PEI–MWCNTs Nanocomposites

#### 2.4.1. FE-TEM Characterization

Solid powder samples of MWCNTs and AuNPs–PEI–MWCNTs were dissolved in water separately to a concentration of 0.25 mg/mL. Thereafter, the solution underwent ultrasonic treatment to produce a uniform and stable dispersion system. The sample was subsequently placed onto a 3 mm copper mesh with a mesh size of 200 and permitted to air-dry at ambient temperature. The procedure was conducted thrice. The shape and structure of the MWCNTs and AuNPs–PEI–MWCNTs were examined using field emission transmission electron microscopy (FE-TEM, Tokyo, Japan, JEM-2100F) with an acceleration voltage of 200 kV and using a ZrO/W (100) Schottky electron gun.

#### 2.4.2. Spectral Characterization of UV-Vis Absorption

Solutions of PEI, AuNPs–PEI, MWCNTs, and AuNPs–PEI–MWCNTs were produced at a concentration of 0.25 mg/mL using ultrapure water. Subsequently, these solutions underwent ultrasonic treatment to attain a stable suspension. The resultant suspensions were subsequently transferred to a cuvette for testing. UV-Vis absorption spectra for PEI, AuNPs–PEI, MWCNTs, and AuNPs–PEI–MWCNTs were acquired through scanning measurements across the wavelength range of 190–800 nm, with a step interval of 1 nm, employing a UV4S02 spectrophotometer (Unico Shanghai Instruments Co., Ltd., Shanghai, China).

### 2.5. Electrochemical Measurements

Electrochemical measurements were carried out utilizing an electrochemical workstation (Chenhua Corp., Shanghai, China) employing a standard three-electrode configuration. The scan range was from −0.2 V to 0.6 V, with a scanning speed of 100 mV/s. The counter electrode was a Pt wire, the reference electrode was an Ag/AgCl electrode, and the working electrode was a modified glassy carbon electrode. All electrochemical measurements were executed in a solution of [Fe(CN)_6_]^3−/4−^ (5 mM, 0.1 M KCl). The electrochemical characteristics of biosensors modified with two materials (MWCNTs and AuNPs–PEI–MWCNTs) were assessed using CV. Furthermore, CV analysis was performed for biosensors at various assembly stages. The Linear Scanning Voltammetry (LSV) technique was utilized to explore the response of the standard samples (Ses i 4 or Ses i 5) to the applied potential, ranging from −0.2 V to 0.6 V, with a scanning rate of 0.1 mV and a dwell time of 2 s per potential. The standard sample was positioned at the center of the modified electrode surface during testing, and the LSV current peak (*I_sample_*) was recorded after a 1 h incubation at 37 °C. Subsequently, the same procedure was conducted using PBS instead of the sample (*I*_0_), and the disparity between the two readings was utilized to determine the concentration (∆*I*, ∆*I* = *I_sample_* − *I*_0_).

### 2.6. Preparation and Measurement of Sesame Ses i 4 and Ses i 5

Since roasting is one of the most important processing technologies for sesame seeds, the roasting procedures of 120 °C and 150 °C for durations of 10, 20, and 30 min, and 180 °C for 10 and 20 min were employed, respectively,. Twenty grams of roasted sesame seeds were then submerged in 180 g of ultrapure water, and the oleosin extraction from white sesame seed was performed according to the previous report with some modifications [24]. After grinding the sesame seed–water mixture, the slurry was passed through a 200-mesh sieve for filtration. Then five grams of sucrose (mass fraction 2.5%) were added and dissolved equally, and the pH of the sample solution was adjusted to 11.0 and then centrifuged at high speed of 10,000× *g* for 30 min. After centrifugation, the upper oil layer was obtained and re-dispersed in an alkaline aqueous solution at pH 11.0, followed by an additional wash. The sesame oil body was mixed with methanol at room temperature for 10 min, and was subsequently centrifuged at 6000× *g* for 10 min. After removing the top methanol phase, the precipitated oil body proteins and the triglyceride layer were collected, and n-hexane in a weight ratio of 1:2 was introduced to solubilize the triglycerides. After centrifugation at 6000× *g* for 10 min, the oil body proteins were obtained and then 0.1 mg of protein was dissolved in 0.1 mM PBS (pH 7.4) buffer containing 2% SDS (5%, *w*/*v*) to obtain 1 mL sample solution for further detection. Then, the sample was diluted to 100-fold (1:100, *v*/*v*) with PBS (0.1 mM, pH 7.4) when used. The commercial ELISA was employed to quantify the concentrations of Ses i 4 and Ses i 5, respectively, as per the guidelines from Mskbio Co., Ltd. (Wuhan, China), by deducing the effects of SDS on the absorbance. Simultaneously, electrochemical methods were employed to assess the amounts of Ses i 4 and Ses i 5, respectively. The results were calculated using calibration curves for Ses i 4 and Ses i 5 standards at 50–400 ng/L concentrations.

### 2.7. Statistical Analysis

At least three independent experiments were performed for each test and data are presented as mean ± standard deviation. Data analysis was performed using Origin 2021 (Origin Lab, Northampton, MA, USA). Significant effects were determined by one-way analysis of variance (ANOVA), with *p*-values < 0.05 considered significant.

## 3. Results

### 3.1. Characterizations of AuNPs–PEI–MWCNTs

**Figure 2:**
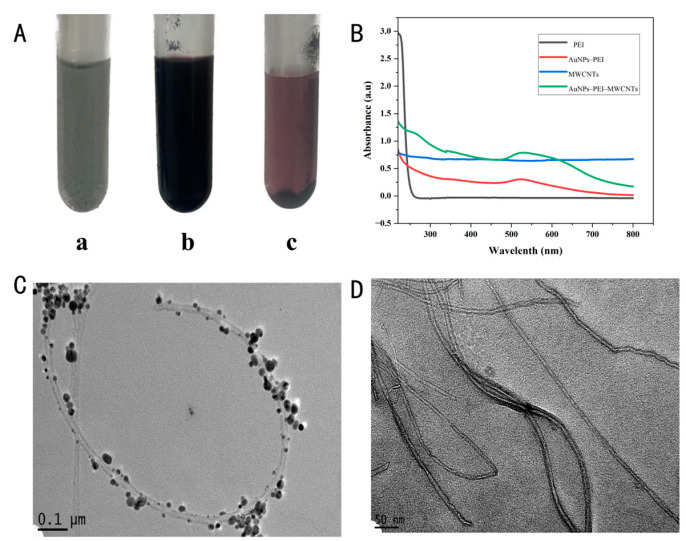
(**A**) Photos of MWCNTs (**a**), PEI–MWCNTs (**b**), and AuNPs–PEI–MWCNTs (**c**), dispersed in distilled water with the concentration of 2 mg/mL. (**B**) UV–Vis spectrum of PEI, AuNPs–PEI, MWCNTs, and AuNPs–PEI–MWCNTs. (**C**,**D**) FE-TEM images of MWCNTs and AuNPs–PEI–MWCNTs.

To enhance electrochemical signals, AuNPs–PEI–MWCNTs were produced. MWCNTs were elected as the principal support material due to their high conductivity, which would promote an improved electron transfer rate and chemical stability. As illustrated in Figure 2A(a), MWCNTs tended to form aggregates, which diminished their dispersion in aqueous solutions. PEI was a cationic polyelectrolyte with many amino groups, and it could efficiently engage with MWCNTs via physical and electrostatic adhesion [16,23,25], which would facilitate a more uniform distribution of MWCNTs, enhancing the structural stability of the composite material. Furthermore, owing to the branching configuration, PEI could function as both a primer and a reducer, facilitating the adsorption of AuCl_4_^−^ ions, which were subsequently reduced to produce AuNPs, and this method facilitated the in situ synthesis of AuNPs on the surface of MWCNTs [26]. Figure 2A(b) illustrates that the effective interaction among AuNPs, PEI, and MWCNTs markedly improved the stability of the composite. UV-spectral analysis (Figure 2B) was conducted to ascertain the successful binding of AuNPs to PEI–MWCNTs. The results indicated that neither PEI nor MWCNTs exhibited absorption peaks within the UV–visible spectrum. Upon bonding with AuNPs, the AuNPs–PEI and AuNPs–PEI–MWCNTs systems exhibited distinct absorption peaks at 525.5 nm, a characteristic wavelength associated with AuNPs in the literatures [27]. This observation confirmed the successful conjugation of AuNPs to the PEI–MWCNTs framework, corroborated by the noticeable shift in the material color to black/dark burgundy (Figure 2A(b)). The presence of carboxyl, amine, thiol, and other functional groups on the surface of AuNPs facilitated the formation of chemical interactions with carbonyl and alkyl functional groups on the surface of MWCNTs, which enhanced the stability of the composite regarding its mechanical, electrical, and chemical properties. The presence of an excessive quantity of AuNPs could lead to suboptimal attachment to PEI–MWCNTs, potentially compromising immunosensor performance. Therefore, it should be imperative to remove any surplus AuNPs. Previous studies have demonstrated that the color intensity of AuNPs was proportional to the concentration [28]. As can be seen from Figure 2A(c), when the concentration was low, the color took on a ruby red, which fully demonstrated that unbound AuNPs could be effectively eliminated.

In order to investigate the surface morphology of AuNPs–PEI–MWCNTs in detail, FE-TEM was used (Figure 2C,D). According to FE-TEM observations, particularly in Figure 2D, the spherical AuNPs were firmly adhered to the MWCNT walls, possessing a diameter between 5 and 20 nm and showed no evidence of aggregation. The MWCNTs displayed a minimal quantity of unbound AuNPs, indicating the successful removal of free AuNPs, which was consistent with the data depicted in Figure 2A(c). In the presence of PEI, AuNPs uniformly dispersed on smaller MWCNTs, providing metal ions and nanoparticles with robust and stable anchoring sites [28]. Consequently, AuNPs–MWCNT composites were successfully synthesized with the assistance of PEI. The surface functional groups of PEI-functionalized MWCNTs exhibited a strong affinity for AuNPs, facilitating their attachment to the nanotube surface. Additionally, PEI served as a co-reactant to amplify the signal. Through the action of PEI, AuNPs were successfully and uniformly distributed across the surface of the MWCNTs. The successful fabrication of these nanocomposites was confirmed through the use of UV-Vis and FE-TEM, which would hold promises for amplifying signals in immunosensor technology applications.

### 3.2. CV Characterization of AuNPs–PEI–MWCNTs

**Figure 3:**
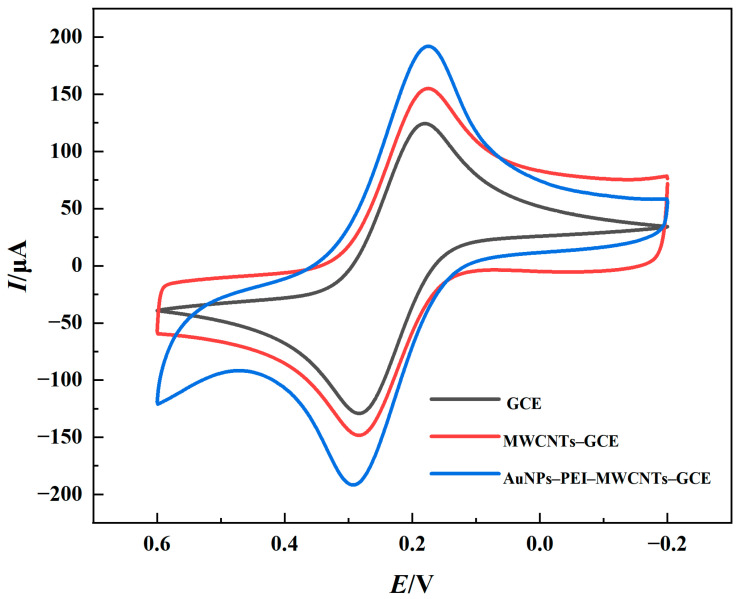
CV characterization of electrodes modified with different materials (GCE, MWCNTs–GCE, and AuNPs–PEI–MWCNTs–GCE).

The modification of electrodes played an important role in the manufacturing of immunosensors. AuNPs–PEI–MWCNTs-modified GCE, bare GCE, and MWCNT-modified GCE were evaluated by CV to examine the contribution of these nanomaterials to electrode signal amplification. As shown in Figure 3, AuNPs and MWCNTs exhibited synergistic effects on electrochemical performance. The difference in potential between the anodic and cathodic peaks of [Fe(CN)_6_]^3−/4−^ solutions on the bare GCE was about 80 mV, indicating that the redox reactions were reversible [29]. The findings showed that the signal from the electrode modified with MWCNTs was much significantly improved, with a notable increase in peak current as indicated by the red line, which might be attributed to the superior electron transport properties of MWCNTs. The CV measurements revealed a substantial increase in peak current when AuNPs were combined with MWCNTs to form an AuNPs–PEI–MWCNTs composite, as shown by the blue line, indicating that the MWCNTs–PEI–AuNPs composite could significantly amplify electrochemical signals and confirming the efficient fabrication of the nanocomposite. An electrode modified with the MWCNTs–PEI–AuNPs composite provided a substantial number of active sites for aptamer attachment, thereby enhancing electronic conductivity [30]. As a result, the composite was found to be highly suitable for signal amplification based on these findings.

### 3.3. CV Characterization of Immunosensor Manufacturing

**Figure 4:**
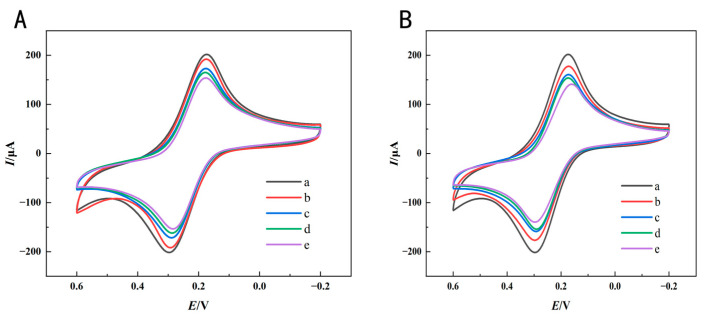
CV characterization of immunosensor for sesame oleosins (**A**) Ses i 4 and (**B**) Ses i 5 at different assembly phases of AuNPs–PEI–MWCNTs (a), SPA–AuNPs–PEI–MWCNTs (b), Ab–SPA–AuNPs–PEI–MWCNTs (c), BSA–Ab–SPA–AuNPs–PEI–MWCNTs (d), and Ses i 4 (Ses i 5)–BSA–Ab–SPA–AuNPs–PEI–MWCNTs (e).

The electrochemical characterization during immunosensor manufacturing was evaluated using CV measurements to determine the viability of the sensing approach. As depicted in Figure 4, a significant enhancement in peak current was noted after the modification using a composite of AuNPs–PEI–MWCNTs, which was ascribed to the superior electronic conductivity of MWCNTs and AuNPs. However, the incorporation of SPA resulted in a reduction in peak current, possibly due to Au-S chemical bonds forming with gold nanoparticles and hindrance effects caused by interactions with biological macromolecules. As a result of the modification of the SPA, electron transfer in the redox pair was obstructive, resulting in a reduction in peak current [30,31]. Furthermore, as the antigen and BSA mixed and gradually adhered to the electrode, resistance values continued to drop, with protein molecules creating a thin barrier to electron transport, thereby hindering electron movement and illustrating the successful incorporation of bioactive materials [32]. Upon the addition of Ses i 4 and Ses i 5 standards, the peak current of the redox signal significantly decreased when these samples interacted with the immunosensor, which could be attributed to the interaction between the antigen surface and the antibody-modified AuNP, facilitating the formation of a thick protein layer. The immunosensor acted as an inert barrier to both electric and mass transfer, preventing ferricyanide from reaching the electrode surface [33]. The charge of proteins might play a relevant role in the redox mechanism of electrochemical probes [34], and the accumulation of charge would lead to the diffusion of redox probes with the same charge being restricted. Therefore, after adding Ses i 4 and Ses i 5 standards, when these samples interacted with the immunosensor, the peak current of the redox signal significantly decreased, which may be attributed to the interaction between the antigen surface and antibody-modified AuNPs, promoting the formation of a thick protein layer [23]. As a result, the immunosensor had been successfully assembled.

### 3.4. Optimization of Detection Conditions

**Figure 5:**
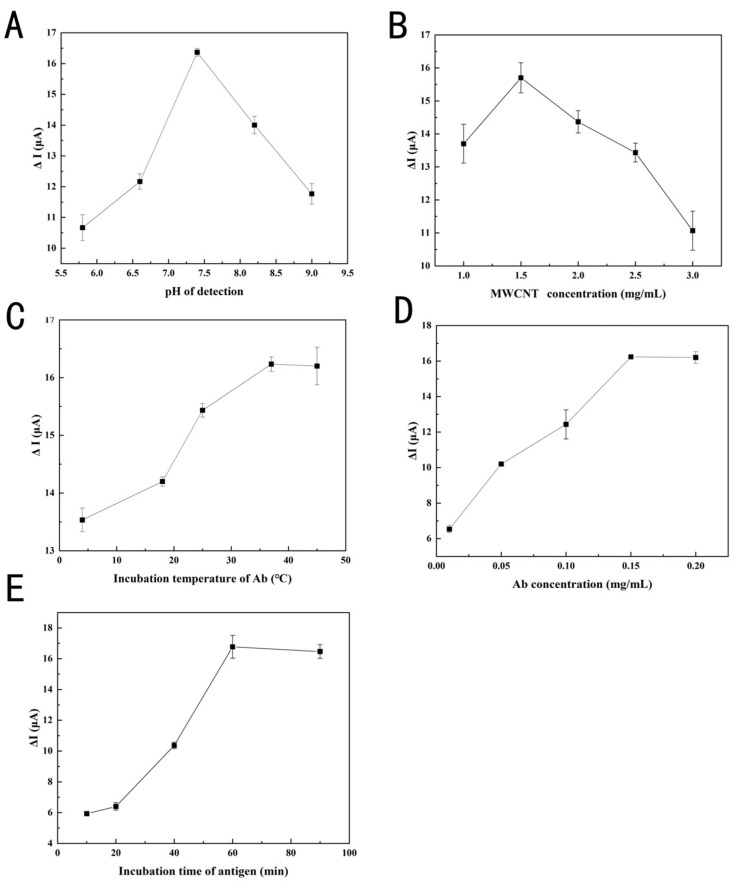
Optimization of detecting parameters for Ses i 4. Effects of (**A**) [Fe(CN)_6_]^3−/4−^ solutions at pH (5.8, 6.6, 7.4, 8.2, and 9.0 in 5 mM, 0.1 M KCl), (**B**) AuNPs–PEI–MWCNTs concentrations (1.0, 1.5, 2.0, 2.5, and 3.0 mg/mL), (**C**) incubation temperature for Ab (4, 18, 25, 37, and 45 °C), (**D**) Ab concentrations (0.01, 0.05, 0.1, 0.15, and 0.2 mg/mL), and (**E**) incubation time (10, 20, 30, 60, and 90 min) for Ses i 4 on the LSV peak current change (Δ*I*). Error bars represent standard deviation, *n* = 3.

**Figure 6:**
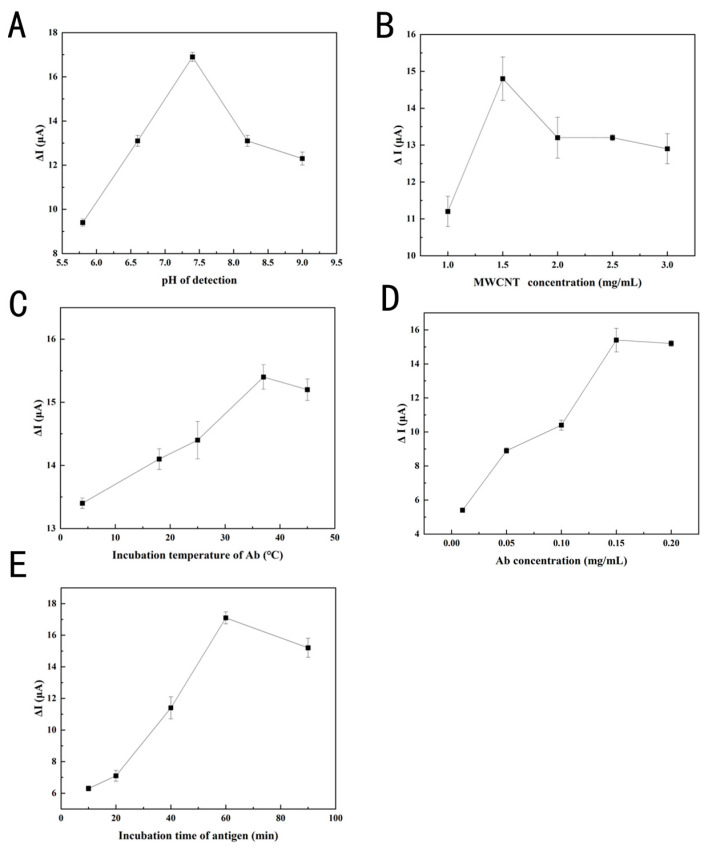
Optimization of detecting parameters for Ses i 5. Effects of (**A**) [Fe(CN)_6_]^3−/4−^ solutions at pH (5.8, 6.6, 7.4, 8.2, and 9.0 in 5 mM, 0.1 M KCl), (**B**) AuNPs–PEI–MWCNTs concentrations (1.0, 1.5, 2.0, 2.5, and 3.0 mg/mL), (**C**) incubation temperature for Ab (4, 18, 25, 37, and 45 °C), (**D**) Ab concentrations (0.01, 0.05, 0.1, 0.15, and 0.2 mg/mL), and (**E**) incubation time (10, 20, 30, 60, and 90 min) for Ses i 4 on the LSV peak current change (Δ*I*). Error bars represent standard deviation, *n* = 3.

To guarantee optimal performance of the immunosensor, the pH of the [Fe(CN)_6_]^3−/4−^ solutions (5 mM, 0.1 M KCl), the concentration of the AuNPs–PEI–MWCNTs composite, the incubation temperature of Ab, and the incubation time of antigen was performed. Extreme pH will adversely affect biomolecules and protein immobilization. In Figure 5A and Figure 6A, the electrical signal intensified at pH 5.8, 6.6, and 7.4, diminished at pH 8.2 and 9.0, and peaked at pH 7.4, indicating that biomolecules needed a neutral environment to stay active. Under neutral conditions, the surface of oleosins became negatively charged as the isoelectric point of oleosins might be in the pH range of 9–10. Concurrently, the synergistic effects of electrostatic repulsion among oleosin molecules and steric hindrance ensured the uniform dispersion of sesame oleosins, preserving their natural structure without disruption [35]. Several experimental results were significantly influenced by AuNPs–PEI–MWCNTs concentration. When the MWCNT concentration was lower than 1.5 mg/mL, the Δ*I* exhibited a linear increase and reached its maximum value at 1.5 mg/mL (Figure 5B and Figure 6B). When the AuNPs–PEI–MWCNTs concentration surpassed 1.5 mg/mL, the electrical signal diminished due to the buildup of agglomeration layers and the detachment of material from the electrode surface [36]. Protein structures were denatured at elevated temperatures (above 40 °C), leading to optimal antibody activity at 37 °C, as depicted in Figure 5C and Figure 6C. Antibodies are normally incubated at 37 °C without further optimization, but after antigen–antibody binding, the incubation period should be carefully considered, as the response sensitivity of the fabricated immunosensor would be directly affected. No significant difference was observed between Figure 5D and Figure 6D when Ab concentration was greater than 0.15 mg/mL. Accordingly, the active Ab binding checkpoint on the SPA has reached its maximum binding capacity. As illustrated in Figure 5E and Figure 6E, the incubation time after antigens were added significantly impacted the interaction between antigens and antibodies. As the incubation duration was prolonged from 10 min to 60 min, the peak current increased and neared saturation. After 60 min, the current remained almost constant, indicating that the maximum binding capacity had been reached, and further increases in incubation time did not yield significant improvements in signal response. Based on optimization, Ses i 4 and Ses i 5 standards in the [Fe(CN)_6_]^3−/4−^ solutions at pH 7.4 could be well detected, respectively, when the AuNPs–PEI–MWCNTs complex concentration was set at 1.5 mg/mL, the Ab concentration was set at 0.15 mg/mL, the Ab incubation temperature was maintained at 37 °C, and the incubation time of antigen was defined at 60 min.

### 3.5. Performance Analysis of Immunosensor

**Figure 7:**
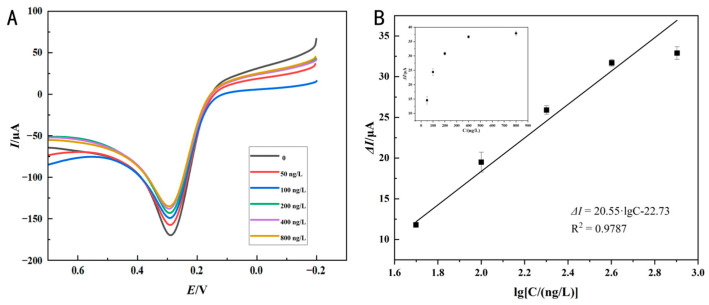
Performance analysis of immunosensor for Ses i 4. (**A**) LSV responses for Ses i 4 standard with the concentrations of 0, 50, 100, 200, 400, and 800 ng/L, in 5 mM [Fe(CN)_6_]^3−/4−^ solutions at scanning rate of 0.1 V/s, (**B**) and corresponding calibration curve (lgC vs. Δ*I*) of immunosensor recorded for 50–800 ng/L Ses i 4; the inset shows the curve of the currents against the Ses i 4 concentrations, error bars represent standard deviation, *n* = 3.

**Figure 8:**
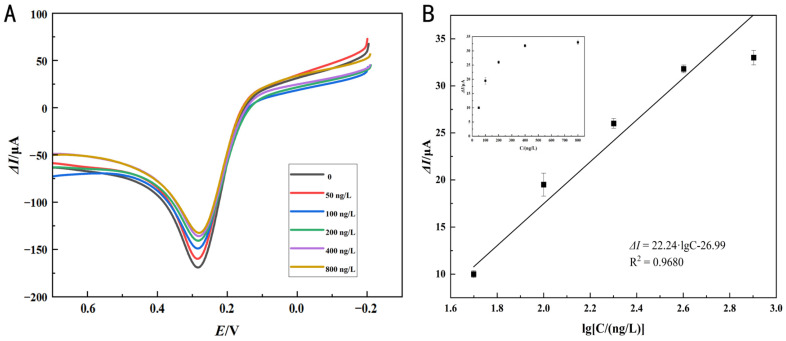
Performance analysis of immunosensor for Ses i 5. (**A**) LSV responses for Ses i 5 standard with the concentrations of 0, 50, 100, 200, 400, and 800 ng/L, in 5 mM [Fe(CN)_6_]^3−/4−^ solutions at scanning rate of 0.1 V/s, (**B**) and corresponding calibration curve (lgC vs. Δ*I*) of immunosensor recorded for 50–800 ng/L Ses i 5; the inset shows the curve of the currents against the Ses i 5 concentrations, error bars represent standard deviation, *n* = 3.

In the optimized conditions, differences in concentrations of sesame oleosins Ses i 4 and Ses i 5 were detected by the LSV method, and the standard curve was calculated based on Δ*I*. As seen in Figure 7A and Figure 8A, when evaluating the analytical performance of the immunosensor for sesame oleosins within the concentration range from 50 to 800 ng/L, it was observed that the LSV response signal decreased with increasing sample concentration, which was primarily due to the insulating effect of the antibody–sesame oleosins immune complex and impeded electron transfer. Although the biosensor responses at the higher concentrations of 400 ng/mL and 800 ng/mL for both Ses i4 and Ses i5 allergens seemed to approach saturation, indicating the maximum detection capacity, further investigation revealed a linear correlation between current amplitude (Δ*I*) and sesame oleosin concentration (the logarithm of the concentration value, lgC) with a linear regression equation of y = 20.55x − 22.73 (R^2^ = 0.9787) for Ses i 4 detection (Figure 7B) and a linear regression equation of y = 22.24x − 26.99 (R^2^ = 0.9680) for Ses i 5 detection (Figure 8B), respectively, suggesting the immunosensor could accurately distinguish between different antigen concentrations. According to the signal-to-noise ratio (S/N = 3), the LOD for Ses i 4 was 0.616 ng/L (Figure 7B) and the LOD for Ses i 5 was 0.307 ng/L (Figure 8B) based on the effective monitoring range of 50–800 ng/L. Additionally, various methods for measuring sesame allergens have been summarized in Table 1. Compared with commercial kits for ELISA assays (8 ng/L), the current work offered the advantage of a lower detection limit and was suitable for real-time food monitoring, which was attributed to its low background current and the complex surface chemistry of the AuNPs–PEI–MWCNTs-modified GCE platform, as the SPA-modified GCE could specifically interact with the Fc region heavy chain of the antibody [37]. Despite LC-MS/MS exhibiting a low detection limit (0.4 and 4 fmol/μL, respectively), the method was costly and intricate to operate [38]. Recent investigations indicated that even minimal consumption of sesame proteins (under 1 mg) would elicit allergic responses [39]. In comparison with the current monitoring methods, the immunosensor created by nanocomposite catalysis and layer-by-layer assembly was able to detect low content of sesame oleosins, thus preventing accidental ingestion by allergic individuals.

### 3.6. Analysis of Anti-Interference Ability, and Stability of Immunosensor

**Figure 9:**
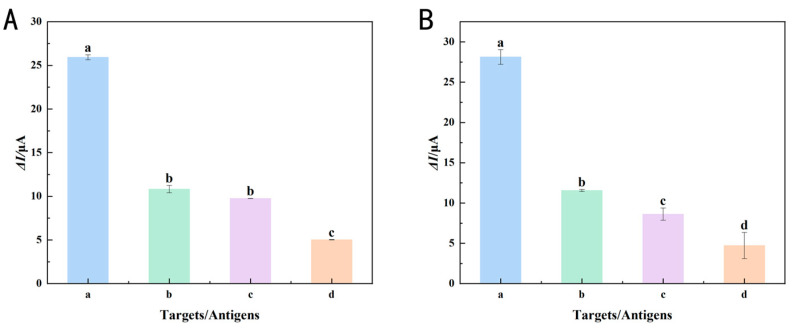
LSV peak current change (Δ*I*) in 5 mM [Fe(CN)_6_]^3−^/^4−^ solution for (**A**) interference study of the immunosensor towards Ses i 4 (a), Ses i 5 (b), black kidney bean lectin (c), and BSA (d); and for (**B**) interference study of the immunosensor towards Ses i 5 (a), Ses i 4 (b), black kidney bean lectin (c), and BSA (d). Error bars represent standard deviation, *n* = 3. Different lowercase letters mean significant differences (*p* < 0.05).

Interference resistance was one of the critical factors for assessing sensor efficacy. After the introduction of 400 ng/L of Ses i 4, black bean lectin, bovine serum albumin, and Ses i 5 were added sequentially to assess the specificity of the immunosensor for Ses i 4, while the Ses i 5 immunosensor was similarly evaluated. Figure 9A,B demonstrated that the Ses i 4 and Ses i 5 immunosensors have considerable selectivity for their corresponding antigens. However, Sesi 4 and Sesi 5 exhibited cross-interference due to their shared origin from the sesame oleosin family with similar molecular weights and analogous structures. The Δ*I* of black bean lectin in the Ses i 4 and Ses i 5 immunosensors was 9.8 and 8.6, respectively, indicating some interference for these potential allergens. Since sesame and beans were both considered oilseed crops, the cross-reactivity should not be ignored. The Δ*I* of the two immunosensors for bovine serum albumin was approximately 5, significantly lower than the Δ*I* of their respective Ses i 4 and Ses i 5, indicating limited selectivity for interfering proteins without homology. The results of this study fully demonstrated that the immunosensor had a highly specific detection capability for the allergenic components of Ses i 4 and Ses i 5, respectively, in practical applications, providing solid technical support for subsequent application development.

**Figure 10:**
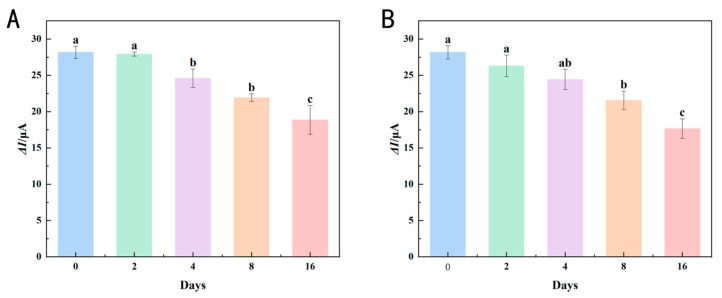
Stability study of the immunosensor for (**A**) Ses i 4 and (**B**) Ses i 5 at days 0, 2, 4, 8, and 16. Error bars represent standard deviation, *n* = 3. Different lowercase letters mean significant differences (*p* < 0.05).

To assess the stability of the immunosensor, it was rinsed with PBS solution and then stored at 4 °C, and the repeatability was tested on days 0, 2, 4, 8, and 16. Figure 10A,B indicate that the Δ*I* for Ses i 4 and Ses i 5 on days 0 and 2 exhibited no significant changes. Nonetheless, from the 4th to the 8th day, Δ*I* diminished, and further declined by the 16th day. The result demonstrated exceptional stability over two days largely due to the effects of MWCNTs. MWCNTs, as a matrix of one-dimensional nanomaterials, had a short ion diffusion length and were less likely to deform under stress, making them robust and durable [46]. Through electrostatic attraction, the AuNPs adhered securely to the sidewalls of the MWCNTs, and because of the adsorptive properties, small particle size, and high surface activity of AuNPs, the immunosensor exhibited stability and biocompatibility, and was resistant to oxidation [12].

### 3.7. Allergenicity Analysis of Ses i 4 and Ses i 5 in Roasted Sample

Sesame seeds are typically consumed in their roasted form, and high-temperature processing can trigger various chemical reactions, including the Maillard reaction, alterations in protein structure, degradation of carbohydrates, and oxidation of lipids, potentially interfering with the allergen detection [47]. Therefore, the impact of food processing on the traits and features of allergens in sesame seeds remained unclear. In this study, the sesame allergens Ses i 4 and Ses i 5 were quantitatively assessed after 120 °C and 150 °C roasting for 10 min, 20 min and 30 min, and 180 °C roasting for 10 min and 20 min, respectively. To study the matrix effect, the calibration curves obtained from standard solutions of 25, 50, 100, 200, and 400 ng/L were compared with the calibration curves of real samples. The real samples were diluted with PBS solution to obtain 100 and then analyzed under optimized conditions. It appeared that the immunosensor had an excellent correlation with ELISA results when the sesame seeds were roasted at 120 °C and 150 °C for short periods (within 20 min) in Table 2. The antibody binding capacity of the sesame oleosins was reduced with the increase in heating intensity. In contrast, the reliability of immunosensor as well as error margin were less than 10%, making the electrochemical immunosensor a reliable, sensitive, and cost-effective technology. It is interesting that further observation showed a significant decrease in Ses i 5 content using the electrochemical immunosensor after high-temperature roasting at 180 °C, leading to discrepancies in the detection outcomes between the immunosensor and ELISA. Compared to the ELISA procedure, the immunosensor could directly detect the sesame oleosins and ignore the influence of SDS. The statistical differences of detection method might be attributed to the destabilization of the Ses i 5 at high temperatures, which would hinder the binding of antigens and antibodies, and a similar phenomenon has been reported for soybean allergen quantification in the 180 °C baking of biscuits [7,48]. Since all detection results fell within the linear range of 50–800 ng/L, the immunosensor in real roasted sesame samples seemed to be applicable. Previous studies have confirmed the increase in protein amount extracted from processed foods with the raising of SDS concentration as the hydrophobic protein content, including denatured or altered proteins, increased in the extraction; then, the higher ELISA results were explainable [49]. Notably, the detection findings of the immunosensor were lower than those of ELISA, also suggesting a higher sensitivity.

## 4. Conclusions

This study introduced a novel nanocomposite material, AuNPs–PEI–MWCNTs, which has been effectively integrated into a glassy carbon electrode to amplify signal intensity, thereby enhancing the detection capabilities for the sesame allergens Ses i 4 and Ses i 5. The exceptional electrical conductivity of the AuNPs–PEI–MWCNTs nanocomposite had endowed the immunosensor with a robust linear response, as evidenced by the determination coefficients (R^2^) of 0.9787 for Ses i 4 and 0.9680 for Ses i 5. The immunosensor boasted a detection range from 50 to 800 ng/L, with impressive limits of detection at 0.616 ng/L for Ses i 4 and 0.307 ng/L for Ses i 5, surpassing the performance of commercially available ELISA kits which typically have a detection limit of 8 ng/L. Furthermore, the immunosensor demonstrated excellent stability, selectivity, and specificity, which were crucial for the accurate identification of sesame oleosins in complex matrices such as roasted sesame samples. This work not only contributed to the advancement of allergen detection but also underscored the potential of AuNPs–PEI–MWCNTs nanocomposites in the field of biosensing. Collectively, our findings presented a promising strategy for the prevention of sesame-induced allergies and introduced a groundbreaking concept for the broader application of these nanocomposites in analytical chemistry. Future work in this area will involve the ongoing optimization of nanocomposite synthesis and a thorough investigation into antibody recognition mechanisms. Efforts will be directed towards refining multiple processes with the goal of minimizing non-specific interactions, thereby enhancing the analytical performance of the next generation of immunosensors.

## Figures and Tables

**Table 1:** Performance comparison of the detection methods for different sesame allergens.

Detection Method	Linear Range	LOD		Sample		Performance Time	References
Immunocolorimetric assay	50–800 μg/L	45.53 μg/L		Sesame proteins		20 min	[40]
Colloidal Gold Immunochromatographic Test Strips	—	1000 μg/ L		Ses i 1 Ses i 2 Ses i 4 Ses i 5		20 min	[41]
Direct ELISA	18.80–300.73 ng/mL	0.15 ng/mL		Ses i 5		5–6 h	[42]
Sandwich ELISA	9.40–150.37 ng/mL	0.37 ng/mL		Ses i 5			
Sandwich ELISA	10–100 ng/L	1 ng/L		Sesame proteins		Overnight + 4 h	[43]
Fluorescence immunoassay	8–640 μg/L	10.15 μg/L		Sesame proteins		20 min	[44]
Hexaplex real-time PCR	—	0.1% (*w*/*w*)		15.5 kDa oleosin gene fragment		NR	[45]
LC-MS/MS	0.1–140 fmol/μL	400 fmol/L		Ses i 4		NR	[38]
		4 fmol/μL		Ses i 5			
Commercial kits	—	8 ng/L	8 ng/L	Ses i 4	Ses i 5	1.5–2 h	Mskbio Co.
Electrochemical immunosensor	50–800 ng/L	0.616 ng/L	0.307 ng/L	Ses i 4	Ses i 5	60 min	This work

**Table 2:** Detection comparison for Ses i 4 and Ses i 5 in roasting samples using immunosensor and ELISA.

Roasting Program	Ses i 4			Ses i 5		
	Immunosensor (ng/L)	ELISA (ng/L)	Δ (ng/L)	Immunosensor (ng/L)	ELISA (ng/L)	Δ (ng/L)
120 °C–10 min	360.60	357.37	3.23	285.44	291.41	−5.96
120 °C-20 min	243.17	243.08	0.09	256.29	255.86	0.43
120 °C-30 min	176.87	179.87	−3.01	199.33	204.65	−5.32
150 °C-10 min	158.22	162.83	−4.61	188.64	192.91	−4.27
150 °C-20 min	148.43	139.63	8.80	144.22	152.38	−8.15
150 °C-30 min	115.44	106.48	8.96	130.72	140.25	−9.53
180 °C-10 min	128.01	125.89	2.11	143.05	155.78	−12.74
180 °C-20 min	102.68	97.96	4.72	116.51	132.30	−15.79

Δ was expressed as the difference between the immunosensor and ELISA measurements, *n* = 3.

## Data Availability

Relevant information and techniques have been provided in this study. The corresponding author should be contacted for any further questions.
